# Proceedings of the 2026 North American Society of Head and Neck Pathology Companion Meeting, San Antonio, TX, March 22, 2026: It’s a Trap! Pitfalls in Thyroid Pathology: Mets or Not Mets?

**DOI:** 10.1007/s12105-026-01901-7

**Published:** 2026-03-14

**Authors:** Yu-Che Chuang, Jen-Fan Hang

**Affiliations:** 1 Department of Pathology and Laboratory Medicine, Taipei Veterans General Hospital, No. 201, Sec. 2, Shipai Rd, Taipei City, 112201 Taiwan; 2 Department of Pathology, School of Medicine, National Yang Ming Chiao Tung University, Taipei, Taiwan; 3 Institute of Clinical Medicine, School of Medicine, National Yang Ming Chiao Tung University, Taipei, Taiwan

**Keywords:** Thyroid, Intranodal thyroid inclusions (ITIs), Papillary thyroid carcinoma (PTC), Metastasis, BRAF VE1, Immunohistochemistry (IHC)

## Abstract

**Background:**

Similar to other head and neck organs, thyroid pathology encompasses a broad spectrum of diagnostic entities. The significant overlap in architectural or cytological features across these entities presents a unique set of diagnostic challenges.

**Methods:**

This review focuses on diagnostic pitfalls related to metastasis in thyroid pathology, with particular emphasis on morphologic assessment and differential diagnosis.

**Results:**

Key scenarios discussed include (1) intranodal thyroid inclusions, which may closely mimic metastatic papillary thyroid carcinoma in cervical lymph nodes, and (2) metastatic tumors to the thyroid that can simulate primary thyroid neoplasms across the full histologic spectrum, from low-grade to high-grade malignancies, especially in the absence of a known concomitant primary malignancy or when the clinical history is not provided. Awareness that both benign thyroid tissue and metastatic tumors may exhibit atypical architectural or cytologic features is critical to avoiding overdiagnosis or misclassification. Rigorous morphological assessment remains the first step in the diagnostic workup. Immunohistochemistry serves as a valuable adjunct when used judiciously, particularly mutation-specific markers such as BRAF VE1 and lineage-associated markers including TTF-1, PAX8, and thyroglobulin, with careful consideration of their sensitivity and specificity.

**Conclusion:**

Integration of histologic findings with clinical history and radiologic information ultimately enables accurate distinction between metastatic and non-metastatic lesions in thyroid pathology.

## Introduction

Similar to other head and neck organs, thyroid pathology encompasses a broad spectrum of diagnostic entities. The significant overlap in architectural or cytological features, not only between non-neoplastic and neoplastic conditions but also across benign, low-risk, and malignant tumors, presents a unique set of diagnostic challenges. One major pitfall concerns benign-looking thyroid follicles found in cervical lymph nodes, known as intranodal thyroid inclusions (ITIs). Although this lesion has been historically controversial, accumulating evidence indicates that not all thyroid follicles within lymph nodes represent metastatic disease. In addition, the potential for ITIs to show architectural or nuclear atypia may lead to overdiagnosis of metastatic thyroid carcinoma. On the other hand, the thyroid gland itself may also serve as a site of metastasis, most commonly from the kidney, lung, and breast. Such metastatic lesions may mimic primary thyroid tumors, including follicular neoplasms or high-grade thyroid carcinomas. Recognition of morphologic “red flags” atypical for primary thyroid neoplasms is therefore crucial. This review examines these diagnostic pitfalls and emphasizes the integration of clinical history and immunohistochemistry (IHC) to ensure accurate diagnosis.

## ITIs Versus Metastatic Thyroid Carcinoma

ITIs refer to benign thyroid follicles in cervical lymph nodes often discovered incidentally during pathological examination of surgical specimens or autopsies. The incidence of ITIs is around 0.7–0.8% in the context of head and neck surgeries with cervical lymph node dissection [1–4], and reaches 4.2% in the context of thyroidectomy with regional lymph node dissection [3]. They were first reported by Frantz et al. in 1942 as “lateral aberrant thyroid tissue in lymph node” in a patient with follicular nodular disease [5], and relevant studies up to 2015 are well documented in a review by Triantafyllou et al. [6]. While some hold onto the conservative view that any thyroid tissue in the lymph nodes should be regarded as metastatic thyroid carcinoma [7, 8], recent studies employing clonality analysis [9], immunofluorescence [10], and IHC [3, 10, 11] have demonstrated that ITIs are different from metastatic thyroid carcinoma in nature and are most likely benign.

Several hypotheses have been proposed to explain the origin of ITIs, including: (1) During thyroid development, the unencapsulated developing thyroid may become mixed with lymph nodes that are also developing in the wall of the jugular lymph sacs [12]; (2) Benign thyroid follicles may migrate away from the thyroid gland to the cervical lymph nodes (so-called “benign metastasis”) [13]; (3) Extrusion of thyroid tissue from the thyroid gland can be surrounded by lymphocytic infiltration, therefore mimicking the picture of thyroid tissue in lymph nodes [13]. One may argue that the term “ITIs” should not be used in the last scenario because lymph node structures should be clearly identified for such terminology. This, however, does not resolve the major challenge of distinguishing benign thyroid follicles in the lymph nodes (or benign extrusion of thyroid gland) from malignant thyroid carcinoma.

### Evolving Diagnostic Criteria for ITIs

In Chap. 1*2: Tumors and Tumor-Like Lesions of the Neck and Lymph Nodes* of the 5th edition of WHO Classification of Head and Neck Tumors, benign thyroid inclusion is listed in a table as a differential diagnosis of heterotopia-associated papillary thyroid carcinoma (PTC), along with several distinguishing features [14]. Similar to prior studies [6, 12, 13], the criteria emphasize the midline/paramidline/central neck location, a bland histologic appearance (“isolated, few or small foci of bland, simple follicles without cytological atypia or architectural complexity”), and a limited extent (“limited to one lymph node”). Also listed is the “absence of radiographic and/or pathological abnormalities in the thyroid gland”, which if applied strictly, would preclude the diagnosis of ITIs in most thyroidectomy specimens. Although the narrow criteria for ITIs intend to avoid underdiagnosis of metastatic thyroid carcinoma, they may conversely lead to an overdiagnosis of metastatic disease and potential overtreatment.

**Fig. 1:**
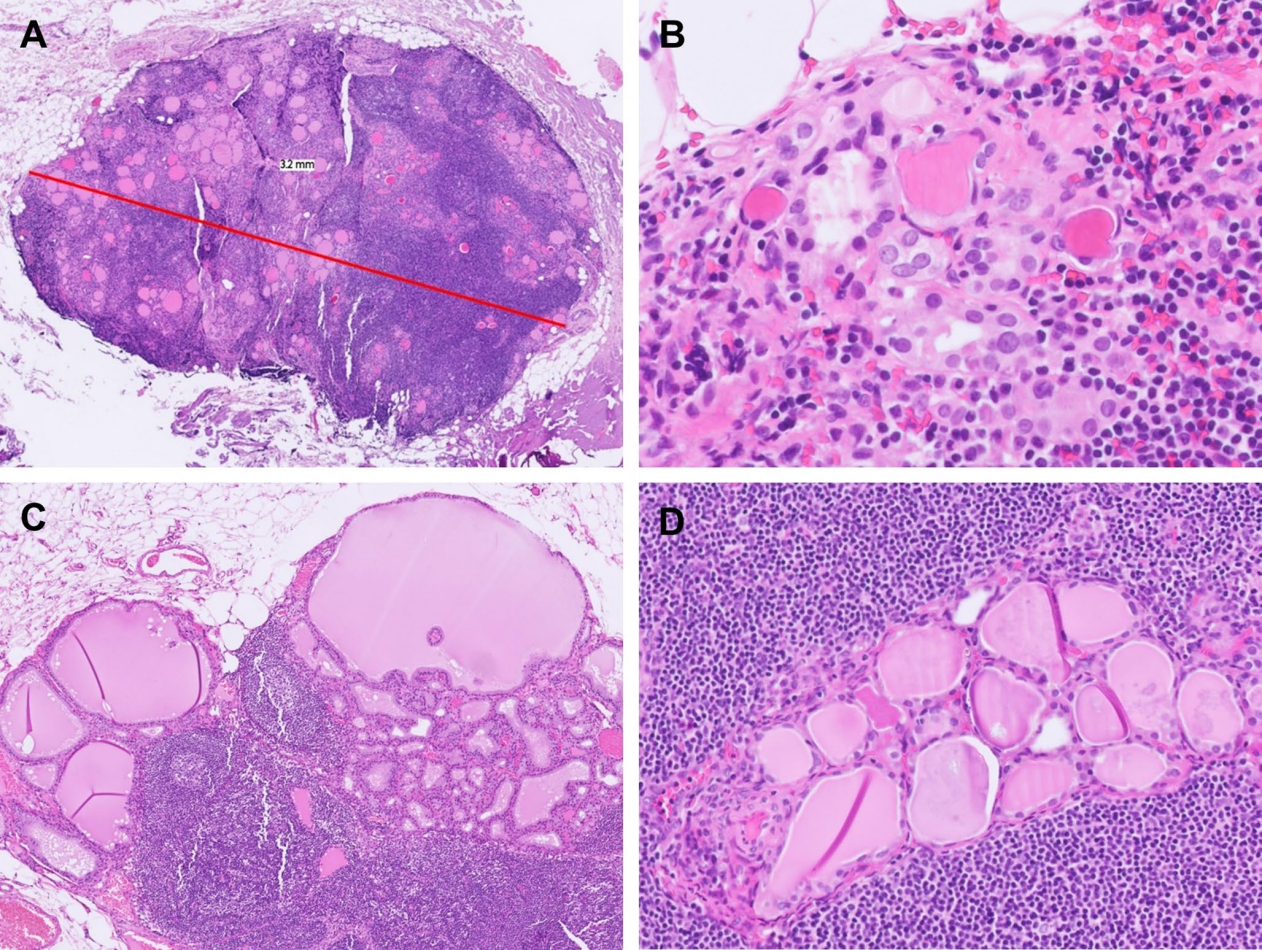
Atypical features of intranodal thyroid inclusions (ITIs). **A** ITIs may reach a substantial size and involve the lymph nodes extensively. This example shows ITIs measuring 3.2 mm in maximal dimension. However, desmoplastic reaction and effacement of lymph node structures are absent. **B** ITIs can exhibit microfollicular architecture and show various degrees of nuclear changes, including enlargement, chromatin alterations, and rare nuclear grooves, similar to those in chronic lymphocytic thyroiditis. **C** ITIs may show hyperplastic papillary infoldings and colloid scalloping in patients with Graves’ disease. **D** Predominantly smooth luminal borders are seen in most ITIs and help differentiate them from metastatic papillary thyroid carcinoma

In fact, the occurrence of ITIs in the lateral neck lymph nodes has been well documented, most commonly in the setting of head and neck surgeries with cervical lymph node dissection [1–3]. In our prior study of consecutive ITI cases, 17% were identified in lateral neck lymph nodes [3]. Moreover, ITIs frequently involve more than one lymph node [12, 15–18]. In our series, 43.9% of cases involved 2 or more lymph nodes, with up to 5 lymph nodes affected [3]. Importantly, several case reports have described ITIs reaching substantial sizes (Fig. 1A) and being radiologically detectable [18–20]. At present, establishing an upper limit for either the number of involved lymph nodes or the maximum acceptable size of ITIs would be arbitrary.

**Fig. 2:**
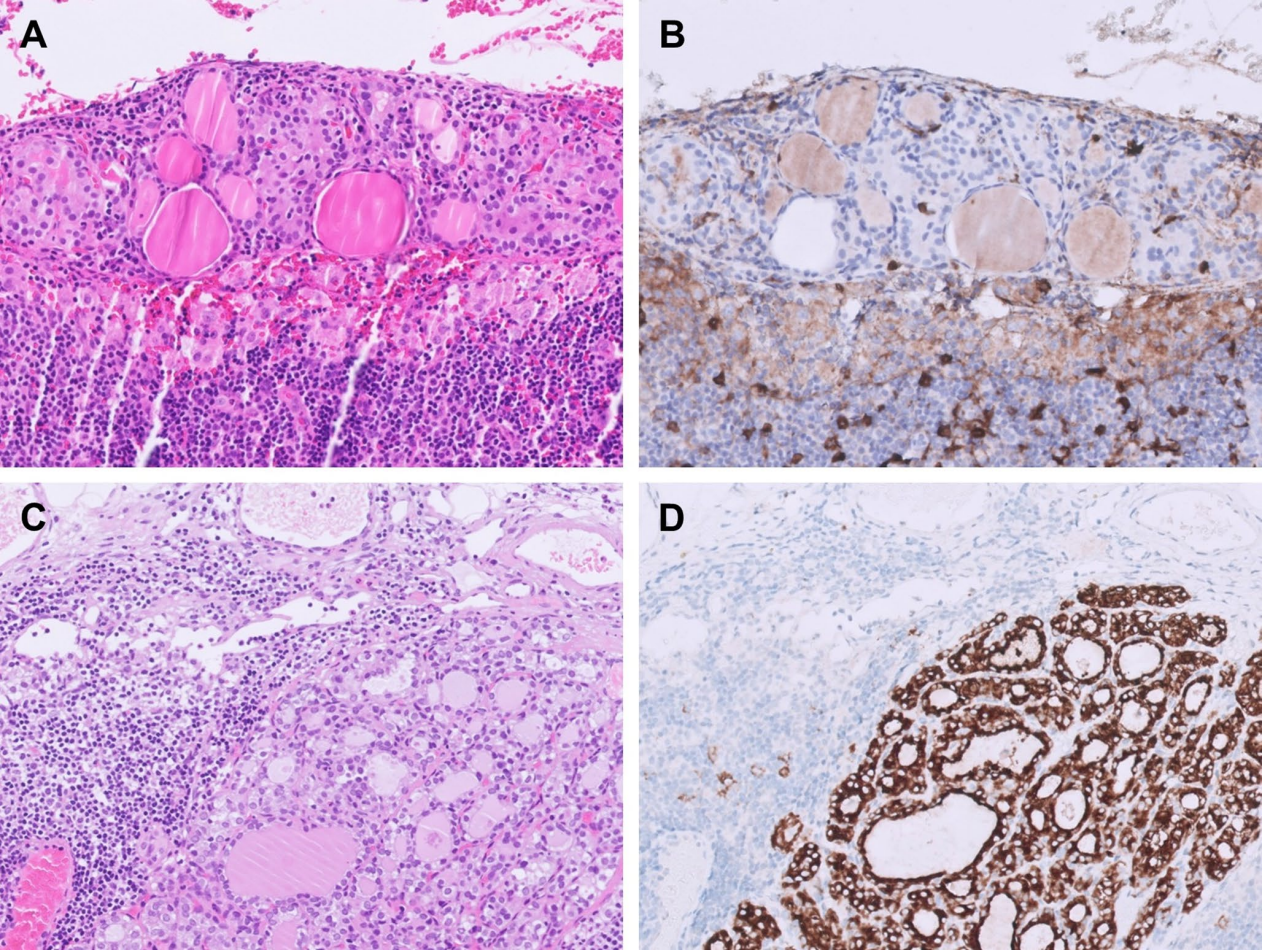
Immunohistochemical features of intranodal thyroid inclusions (ITIs) versus metastatic papillary thyroid carcinoma (PTC). **A** A focus of subcapsular ITIs shows microfollicular architecture with slightly enlarged nuclei. **B** The ITIs are negative for HBME-1 (shown here) as well as galectin-3 and BRAF VE1 (not shown). **C** A lymph node metastasis of *ALK*-rearranged PTC, demonstrating thyroid follicular cells with smooth luminal borders and subtle nuclear atypia, mimicking ITIs. **D** The tumor cells are diffusely positive for ALK (D5F3) immunohistochemistry

With regard to the morphological features of ITIs, Otsubo et al. reported 2 cases of thyroid inclusions with questionable PTC-like nuclear features, but both were negative for BRAF V600E, HBME-1, and galectin-3 IHC as well as TP53-binding protein 1 (53BP1) immunofluorescence, supporting the authors’ interpretation of these lesions as true ITIs [10]. We corroborated these observations and further demonstrated that ITIs may exhibit atypical morphology such as chromatin alterations and nuclear grooves (Fig. 1B), simulating reactive changes seen in chronic lymphocytic thyroiditis [3]. Additionally, in patient with Graves’ disease, histologic changes consistent with diffuse hyperplasia may also be observed in ITIs, likely reflecting the systemic effect of circulating autoantibodies (Fig. 1C). In our experience, the most useful diagnostic features to distinguish ITIs from metastatic *BRAF* p.V600E-positive PTC are the absence of nuclear pseudoinclusions and the presence of predominantly smooth luminal borders (Fig. 1D) [3]. In addition, although the nuclei of ITIs may appear enlarged, they are usually smaller than those of metastatic PTC. Negative BRAF VE1, HBME-1, and galectin-3 IHC further support the diagnosis of ITIs (Fig. 2A, B). It should be acknowledged, however, that HBME-1 and galectin-3 IHC may show focal staining in non-neoplastic thyroid tissue or benign thyroid tumors, and that due to laboratory-dependent variability in staining quality, interpretation of these two markers requires caution. While BRAF VE1 is very useful for distinguishing ITIs from *BRAF* p.V600E-positive metastatic PTC, it is not informative in distinguishing ITIs from metastatic PTC harboring other molecular drivers, such as *RET*,* NTRK*, or *ALK* fusions. We recommend adding additional immunohistochemical markers (e.g. pan-TRK, ALK, etc.) if the specific genetic alteration of the primary thyroid carcinoma is known to the examining pathologist (Fig. 2C, D). Similarly, interpretation of these next-generation markers requires experience to avoid misinterpreting artifactual or nonspecific staining as true positivity [21]. In cases of intranodal thyroid tissue with ambiguous morphology and equivocal immunostaining results, molecular testing may ultimately be considered to aid in definitive classification. However, given the often limited size of these lesions, one must remain mindful of the potential limitations of low tumor purity and the risk of false-negative results.

As a side note, given that lymph node metastasis of follicular thyroid carcinoma (FTC) is exceedingly rare, the discovery of bland intranodal thyroid tissue in a case with primary FTC necessitates the consideration of ITIs. In the absence of a more high-grade component in the intranodal thyroid tissue, distinguishing metastatic FTC from ITIs based on morphology is extremely difficult if not impossible. Furthermore, it remains unclear whether the rare historical reports of nodal FTC metastasis shared the same molecular profile as the primary FTC, or were in fact ITIs over-interpreted as metastatic disease. In cases where the primary FTC is positive for RAS Q61R IHC, this stain may serve as a valuable tool to clarify the nature of the concurrent intranodal thyroid tissue.

### Coexistence of ITIs and Metastatic Thyroid Carcinoma

**Fig. 3:**
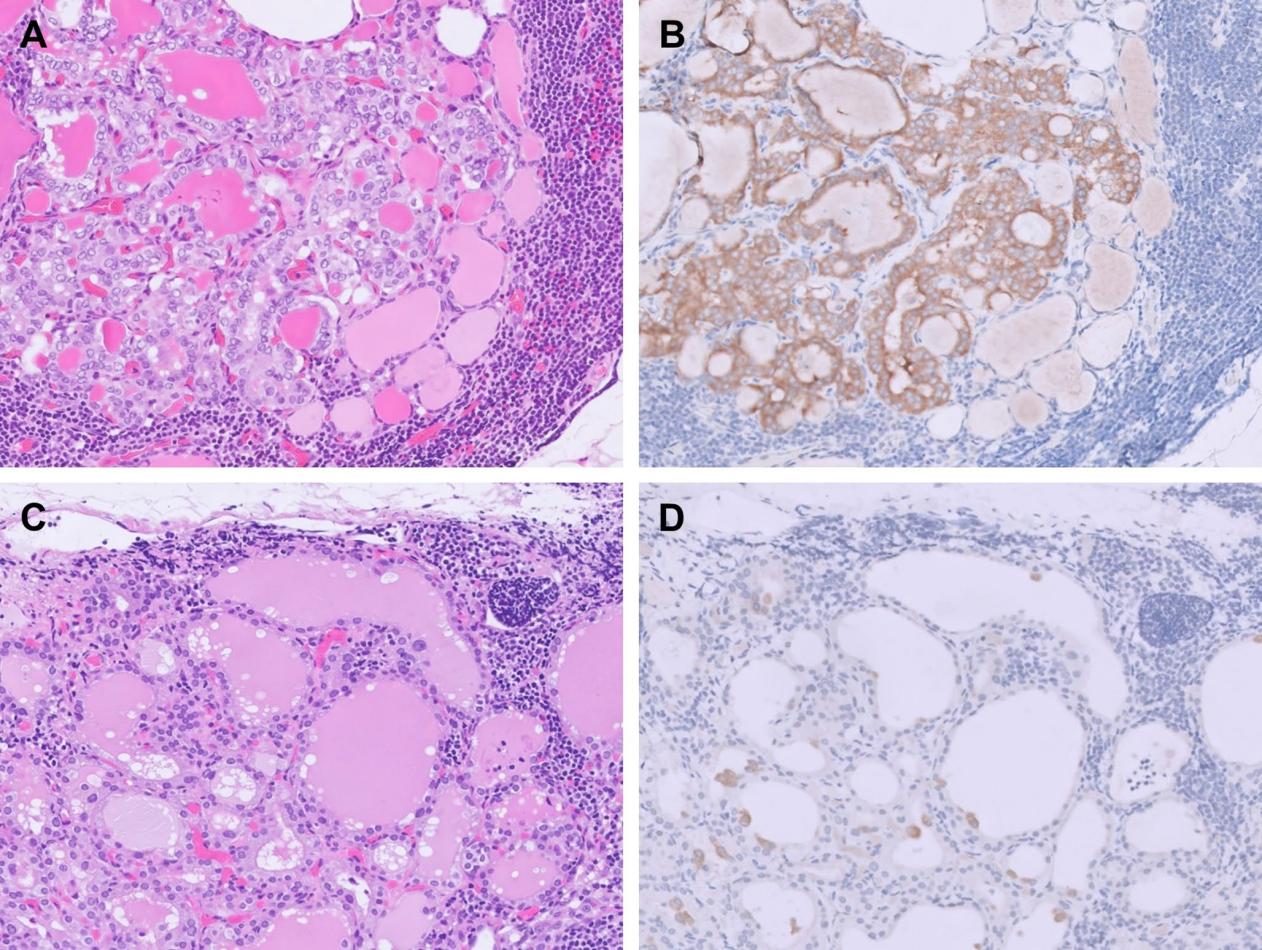
Coexistence of metastatic papillary thyroid carcinoma (PTC) and intranodal thyroid inclusions (ITIs) within the same lymph node. **A** Metastatic PTC intimately intermixed with ITIs. **B** Metastatic PTC highlighted by BRAF VE1 immunohistochemistry. **C** Scattered single cells or small clusters of metastatic PTC are present on the follicular wall of ITIs, which may be easily overlooked on routine morphologic examination. **D** BRAF VE1 immunohistochemistry clearly highlights the metastatic PTC cells

In the study by Otsubo et al., coexistence of ITIs and metastatic PTC in the same lymph node was mentioned but not further discussed [10]. We subsequently demonstrated that such coexistence is not uncommon, and we hypothesized that it may result from PTC cells accessing the same lymphatic route as thyroid inclusion cells or reflect a form of “metastasis organotropism” [3, 22]. The coexistence can be classified into 3 patterns: (1) metastatic PTC and ITIs at separate foci; (2) metastatic PTC and ITIs closely apposed or intermixed with each other (Fig. 3A, B); and (3) metastatic PTC presenting as isolated single cells or small clusters on the follicular wall of ITIs (Fig. 3C, D). The coexistence patterns may change with deeper levels of sectioning, and awareness of these patterns minimizes the risk of overlooking true metastatic disease.

### Clinical Implications of Recognizing ITIs

In thyroid cancer specimens, the stringent distinction between intranodal thyroid inclusions and metastasis in every lymph node is not always required. However, awareness of the atypical morphologic features of ITIs, along with recognition of their coexistence with metastatic PTC in the same lymph node, will ensure accurate enumeration of metastatic lymph nodes and measurement of metastatic foci. This, in turn, ensures more precise recurrence risk stratification under the 2025 American Thyroid Association management guidelines, in which pN1a disease (≤ 5 lymph nodes, all ≤ 2 mm) is categorized as low risk, with direct implications for subsequent clinical management [23].

When evaluating specimens of other head and neck cancers, pathologists may incidentally encounter thyroid follicular cells within cervical lymph nodes that exhibit architectural or nuclear atypia without overt malignant features. In such situations, utmost caution is warranted, as ITIs themselves may also show varying degrees of atypia. For a definite diagnosis of metastatic thyroid carcinoma, more specific features (e.g. nuclear pseudoinclusions, undulating luminal borders, desmoplastic reaction, effacement of lymph node structures) should be looked for, with ancillary IHC (e.g. BRAF VE1, HBME-1, and galectin-3) performed when appropriate. If BRAF VE1 IHC is negative but the nature of the intranodal thyroid tissue remains uncertain owing to its atypical morphologic features, lateral neck location, or equivocal HBME-1/galectin-3 staining result, a descriptive diagnosis of “intranodal thyroid tissue” would be justified. This may be accompanied by a cautionary note explaining that due to the overlapping features of benign ITIs and metastatic thyroid carcinoma, a thorough clinical examination of the thyroid is needed to confidently exclude an occult thyroid primary, and that if a primary tumor is subsequently discovered, correlation of its morphology and BRAF VE1 profile with the intranodal thyroid tissue is needed for an accurate pathological staging. Clear communication with the treating clinician is essential to avoid unnecessary thyroidectomy in the absence of a confirmed primary lesion.

## Metastasis to the Thyroid Versus Primary Thyroid Tumor

Metastasis to the thyroid is uncommon and presents diagnostic pitfalls to pathologists not wary of this possibility. In autopsy series, the overall incidence of metastasis to the thyroid is 2%, with the most common sites of origin being the lung and breast [24]. In clinical series, metastasis to the thyroid accounts for 0.36–3% of all thyroid malignancies, and the most common sites of origin are the kidneys (often clear cell renal cell carcinoma [RCC]), followed by the lungs, breast, head and neck, and gastrointestinal tract [24–28]. Many of such cases have a known cancer history, but occasionally, metastasis to the thyroid is the initial manifestation of an underlying malignancy [25, 26, 29]. The time from diagnosis of primary cancer to metastasis to the thyroid gland can also be prolonged, notably for RCC with a median interval of around 9 years [26, 30, 31]. Moreover, a non-thyroid malignancy may rarely metastasize to a primary thyroid tumor or adenomatous nodule, further complicating the pathologic diagnosis [25, 32, 33].

**Fig. 4:**
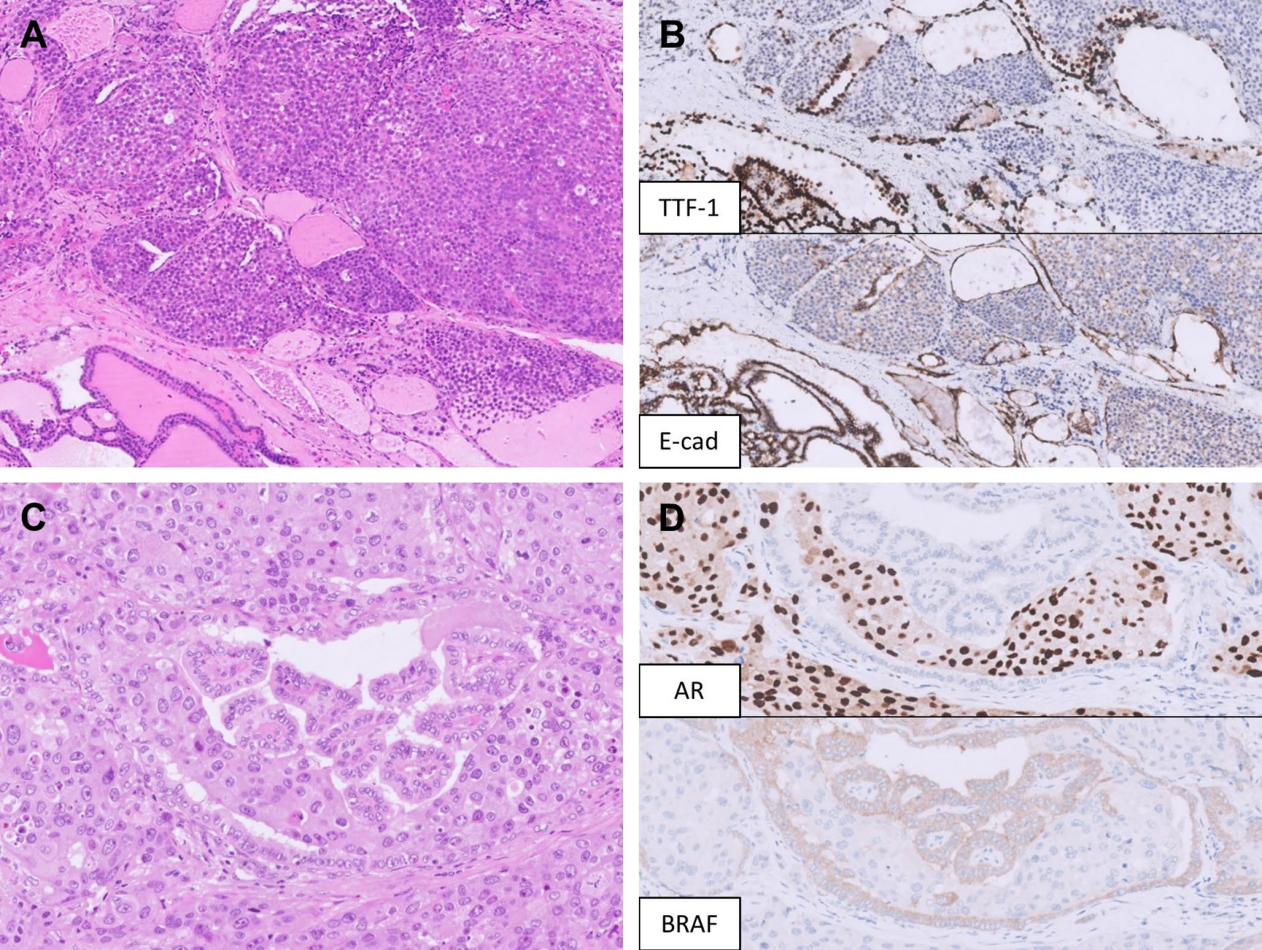
Metastatic malignancies mimicking primary thyroid carcinoma. **A** A metastatic invasive lobular carcinoma of the breast mimics an oncocytic thyroid carcinoma. **B** The tumor cells are negative for TTF-1 and show loss of E-cadherin. **C** A salivary duct carcinoma (SDC) metastasizes to a papillary thyroid carcinoma (PTC), mimicking anaplastic transformation. **D** AR and BRAF VE1 immunohistochemistry stain the SDC and PTC components, respectively

Table 1 shows cases of metastasis initially diagnosed as primary thyroid tumors reported in recent literature. The most common pitfalls are (1) diagnosing metastatic RCC as primary follicular neoplasms, and (2) diagnosing metastatic carcinoma (often lung adenocarcinoma) as poorly differentiated thyroid carcinoma (PDTC) or anaplastic thyroid carcinoma (ATC). Metastasis to the thyroid can be suspected based on careful pathological examination. As a rule, predominantly solid, cribriform, and micropapillary growth patterns without follicle formation or colloid secretions are unusual for common follicular cell-derived neoplasms (Fig. 4A, B). Additionally, marked pleomorphism, prominent nucleoli, and other high-grade features, including increased mitoses and necrosis, also raise suspicion of metastasis [34–36]. While high-grade features in the thyroid often prompt consideration of high-grade thyroid cancers, such as high-grade differentiated thyroid carcinoma, PDTC, and ATC, it is important to exclude metastasis before diagnosing these rarer primary thyroid tumor entities, especially in the context of tumor-to-tumor metastasis (Fig. 4C, D).

**Table 1:** Reported cases of metastases initially misdiagnosed as primary thyroid tumors in recent literature

	Specimen type	Number of cases	Initial diagnosis	Final diagnosis
Pusztaszeri et al. [36]	FNA	3	Suspicious for follicular neoplasm	RCC
		2	1 PTC, 1 poorly differentiated thyroid carcinoma	Adenoid cystic carcinoma
		1	Malignant: high-grade carcinoma (papillary versus anaplastic)	SCC
Hegerova et al. [26]	FNA	6	Follicular neoplasm, PTC	RCC, lung carcinoma
Kanjanahattakij et al. [45]	FNA	1	Poorly differentiated thyroid carcinoma	Lung adenocarcinoma
Prameela et al. [49]	Surgical specimen	1	Poorly differentiated carcinoma	Lung adenocarcinoma
Battistella et al. [48]	FNA	2	PTC	RCC
		1	Poorly differentiated thyroid cancer	Colorectal cancer
Ghossein et al. [25]	Surgical specimen	3	ATC	1 lung non-small cell carcinoma, 1 *EML4*::*ALK* lung adenocarcinoma, 1 leiomyosarcoma
		1	PTC follicular variant	Chromophobe RCC
Stergianos et al. [30]	FNA	2	Suspicious for follicular neoplasm	Clear cell RCC
		1	ATC	Poorly differentiated lung adenocarcinoma
Velez Torres et al. [38]	FNA	1	Suspicious for follicular neoplasm	Clear cell RCC

ATC, anaplastic thyroid carcinoma; FNA, fine needle aspiration; PTC, papillary thyroid carcinoma; RCC, renal cell carcinoma; SCC, squamous cell carcinoma

### Metastasis Mimicking Well-Differentiated Thyroid Tumors

Metastatic clear cell RCC, with low-grade to intermediate-grade nuclear features and abundant clear to eosinophilic cytoplasm, may resemble an oncocytic thyroid neoplasm or a follicular thyroid neoplasm with clear cell change, especially on FNA cytology [29, 35, 36]. In surgical specimens, metastatic clear cell RCC often exhibits a prominent sinusoidal vasculature, further mimicking a follicular thyroid neoplasm. Wood et al. [37] reported that follicular thyroid neoplasms tend to maintain some cytoplasmic granularity, and the absence of glycogen and lipids also points towards a follicular thyroid neoplasm. Velez Torres et al. [38] found that metastatic clear cell RCC is often characterized by a prominent thick fibromuscular pseudocapsule with thick malformed vessels and myxoid change, distinct from a primary thyroid tumor, which is usually surrounded by a thin fibrous pseudocapsule. Since clear cell RCC and follicular thyroid neoplasms often express PAX8, additional immunohistochemical stains such as TTF-1, thyroglobulin, and CA9 are useful in establishing the correct diagnosis. A profile of TTF-1 (−)/thyroglobulin (−)/CA9 (+) was previously shown to be 100% sensitive and 100% specific for diagnosing metastatic clear cell RCC [39]. Importantly, CK7 is insufficient for distinction, since clear cell RCC may focally but strongly express CK7 [40, 41].

**Fig. 5:**
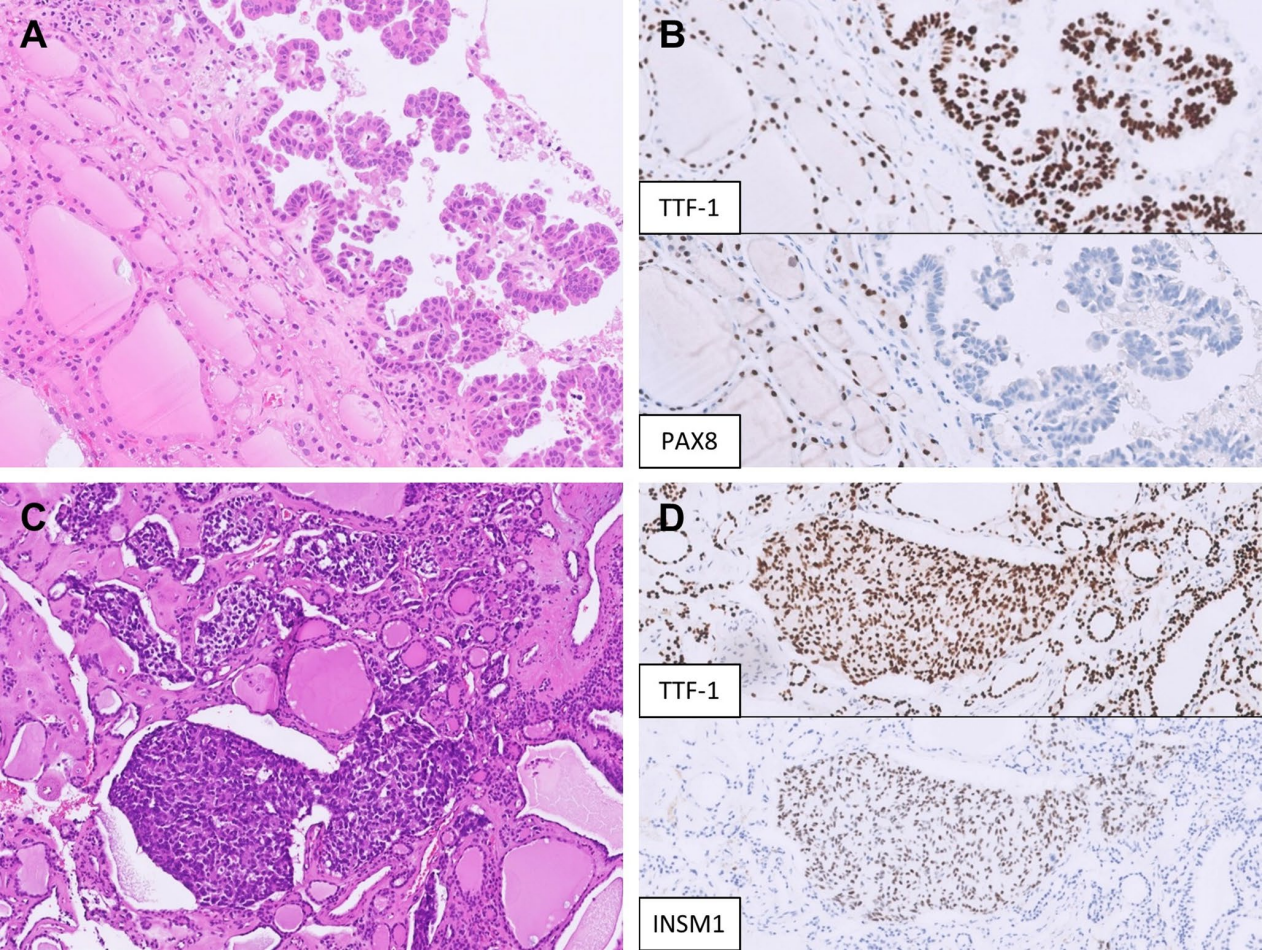
Metastatic malignancies mimicking low-grade thyroid carcinoma. **A** A metastatic lung adenocarcinoma shows a predominant papillary and focal micropapillary growth pattern with oval nuclei, occasional grooves, and psammoma bodies. **B** The tumor cells are positive for TTF-1 but negative for PAX8. **C** A metastatic neuroendocrine tumor of unknown primary presents with multifocal solid nests within an adenomatous thyroid nodule, mimicking medullary thyroid carcinoma. **D** The tumor cells are diffusely positive for TTF-1 and INSM1 (shown here) but negative for calcitonin and CEA (not shown)

Metastatic lung adenocarcinoma may present with a papillary growth pattern with nuclear elongation, occasional grooves or pseudoinclusions, thereby mimicking PTC (Fig. 5A, B). However, architectural patterns that are unusual for classic PTC are often observed, including acinar, micropapillary, complex glandular, cribriform, or solid growth patterns [42], frequently accompanied by pleomorphic nuclei [32]. Additionally, true neoplastic follicles containing colloid are absent in metastatic lung adenocarcinoma [43]. Since TTF-1 is expressed by both lung adenocarcinoma and PTC, PAX8 and thyroglobulin are the two most reliable markers that support a thyroid primary. Importantly, polyclonal PAX8 antibodies show rare positivity in lung adenocarcinoma (2.4%). This can be addressed by using monoclonal PAX8 antibodies, which provide better specificity [44]. To distinguish between PTC and metastatic lung adenocarcinoma, thyroglobulin is highly sensitive and specific for PTCs [43]. However, its sensitivity may be compromised in limited tissue samples where expression is more focal or weaker compared to surrounding normal thyroid [43]. On the other hand, false positivity can rarely occur due to tissue contamination by thyroglobulin-rich colloid from adjacent thyroid parenchyma [43]. This was highlighted in a case report where metastatic lung adenocarcinoma showed heterogeneous thyroglobulin staining [45]. Napsin A, which is commonly used in the diagnosis of lung adenocarcinoma, is less useful in this context, as it is expressed in up to 30.3% of PTCs [46].

On another note, the presence of nested, insular, or trabecular architecture accompanied by mild to moderate nuclear atypia with neuroendocrine features could suggest medullary thyroid carcinoma (MTC). However, co-expression of TTF-1 and neuroendocrine markers is not sufficient to distinguish MTC from metastatic neuroendocrine neoplasms, as TTF-1 may also be expressed in pulmonary and extrapulmonary neuroendocrine carcinomas as well as in some lung carcinoids (Fig. 5C, D) [47]. A definitive diagnosis of MTC therefore requires additional immunohistochemical confirmation with calcitonin and/or CEA, together with correlation with the patient’s clinical history and laboratory findings.

### Metastasis Mimicking High-Grade Thyroid Carcinomas

Another major pitfall is misdiagnosing a metastatic carcinoma as a primary high-grade thyroid carcinoma [25, 30, 36, 48, 49]. When a metastatic poorly differentiated carcinoma shows solid, trabecular, or sheet-like growth pattern with marked nuclear atypia, it may be a great mimicker of PDTC or ATC. In this setting, PAX8 and TTF-1 IHC are more helpful than thyroglobulin which is usually negative in ATCs [25, 50]. Polyclonal PAX8 antibodies are positive in 70–79% of ATCs [50–52], while remaining negative in the majority of lung carcinomas. Rare positivity has been reported in lung adenocarcinoma (2.4%) and lung squamous cell carcinoma (1.9%) [44]. In contrast, a large-scale study found that the commonly used monoclonal PAX8 antibody (MRQ-50) stained only 54.4% of ATCs [53]. Due to the reduced sensitivity of monoclonal PAX8 antibodies to detect ATCs, the selection of a monoclonal or polygonal PAX8 antibody should be decided based on the clinical context and awareness of the caveats. In certain cases, the combined use of monoclonal and polyclonal PAX8 IHC may increase diagnostic confidence in identifying ATC and excluding metastatic lung carcinoma.

**Fig. 6:**
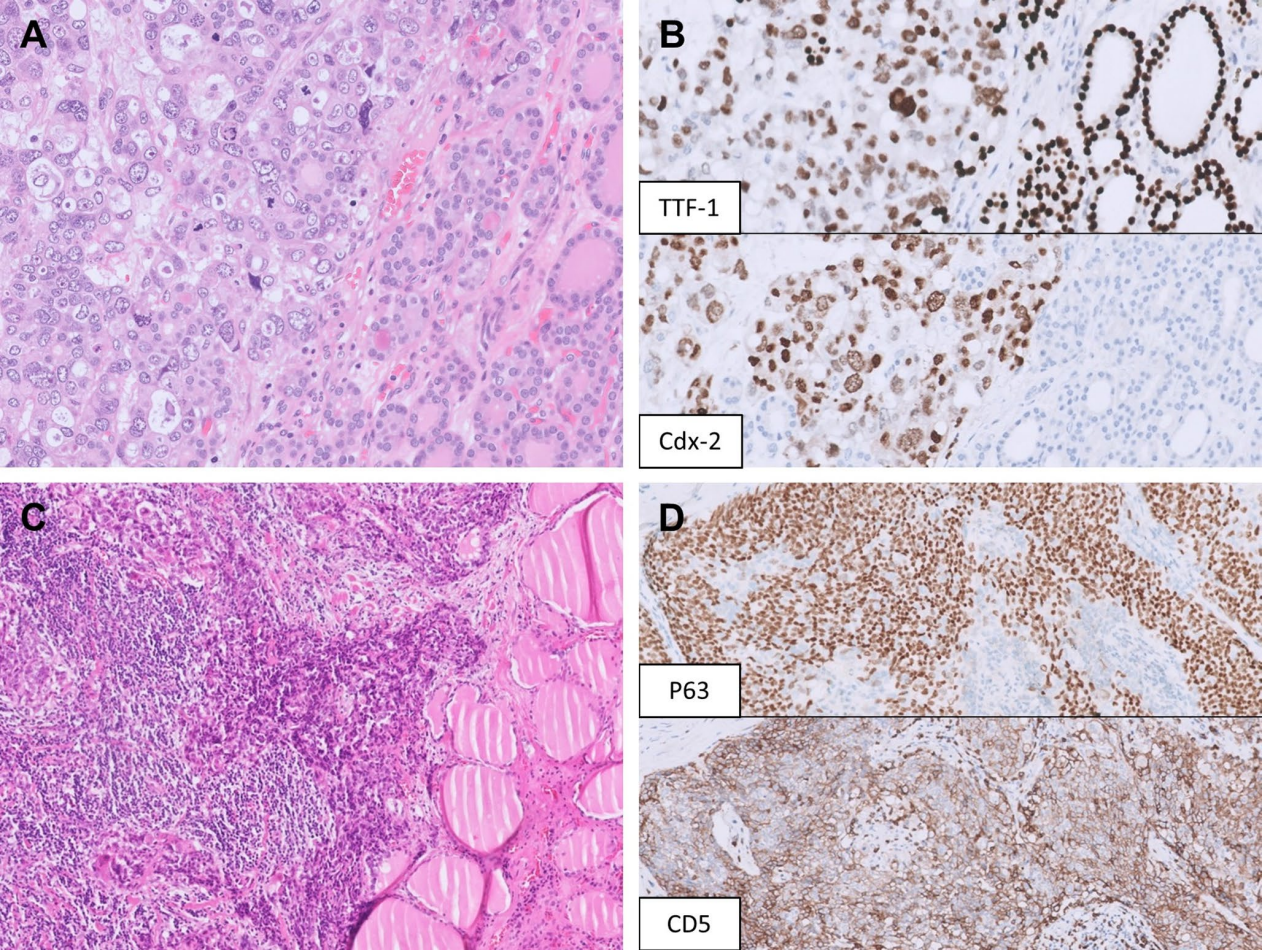
Metastatic malignancies mimicking high-grade thyroid carcinoma. **A** A metastatic adenocarcinoma from the gastroesophageal junction involves a follicular adenoma, simulating high-grade transformation. The metastatic adenocarcinoma shows marked nuclear pleomorphism and cribriform growth pattern without colloid formation. **B** The metastatic adenocarcinoma shows diffuse, moderate TTF-1 expression, an unusual finding; however, CDX-2 expression confirms its gastroesophageal origin. Retrospective TTF-1 immunohistochemistry performed on the patient’s prior endoscopic biopsy of the gastroesophageal adenocarcinoma is also positive. **C** An invasive thymic carcinoma arising from the upper mediastinum was initially diagnosed as anaplastic thyroid carcinoma with squamous differentiation at an outside institution in a young woman presenting with an anterior neck mass. Review of all slides suggests that the tumor is not centered within the thyroid gland. **D** The tumor cells show diffuse p63 positivity; however, co-expression of CD5 (shown here) and CD117 supports a thymic origin

A recent study found that SATB2 was detected in 74% of ATCs, and that 88% (7/8) of ATCs without monoclonal PAX8 expression were positive for SATB2 [54]. However, SATB2 can be positive in various cancers [55, 56] and plays a role in epithelial-mesenchymal transition or cancer progression across different tumor types [57, 58]. Therefore, SATB2 cannot be used alone to differentiate ATC from metastasis to the thyroid. TTF-1 is negative in most ATCs (70–87%) [50, 52, 54], and a positive TTF-1 result (especially when strong and diffuse) in a ATC should prompt other ancillary studies and checking with the clinical history to exclude metastatic carcinoma. Importantly, TTF-1 is occasionally positive in adenocarcinoma from the colorectum, gynecologic tract, and prostate amongst other organs (Fig. 6A, B), especially with clone SP141 and SPT24 that tend to be less specific than clone 8G7G3/1 [59–63].

Another diagnostic challenge arises in the evaluation of thyroid tumors with squamous differentiation. The initial step is to exclude inflammatory conditions, such as chronic lymphocytic thyroiditis, as well as reactive changes associated with fibrosis, hemorrhage or a prior procedure. Once malignancy has been established, it is important to keep in mind that involvement by metastasis or local extension from nearby cancers (especially laryngeal or pharyngeal cancer due to anatomical proximity) may be slightly more common than primary thyroid carcinoma with squamous differentiation (most commonly PTCs, followed by ATCs) [64]. Examination of the tumor border and its relation to the thyroid capsule is crucial to determine if the tumor is centered outside the thyroid and provides important clues of secondary involvement. Thorough sampling will help establish a primary thyroid origin, since a differentiated component can be found in up to half of ATC cases [50, 64], and the frequency may be even higher for ATCs with a pure squamous phenotype [50]. For a limited biopsy specimen, PAX8, TTF-1, and mutation-specific markers (e.g. BRAF VE1, RAS Q61R) are useful for diagnosing an ATC, but negativity for all these immunohistochemical stains does not exclude an ATC. A warning sign against ATC diagnosis is when a tumor expresses p40 or p63 supporting a squamous phenotype, has lost both PAX8 and TTF-1 expression, but does not exhibit marked nuclear pleomorphism. In this scenario, secondary involvement by head and neck squamous cell carcinoma (SCC) as well as thymic-type neoplasms (either primary intrathyroidal or involvement from the thymus) should both be considered (Fig. 6C, D). A recent study on primary thyroid SCCs and ATCs demonstrated that none of the cases expressed CD5 or CD117 [65]. Expression of either marker favors a diagnosis of thymic-type squamous cell carcinoma, with CD5 showing better specificity than CD117 [66].

For rare cases of metastasis to the thyroid without prior history, pathologists may refer to other comprehensive reviews outlining diagnostic approaches using IHC for carcinomas of unknown primary site [67, 68]. In difficult cases, molecular studies can provide additional information regarding tumor origin and guide treatment planning [69]. Nevertheless, the initial and most critical step is the identification of histologic “red flags” which, when correlated with the patient’s clinical history, guide the selection of targeted immunohistochemical panels for a definitive diagnosis of secondary thyroid tumors.

## Conclusion

In summary, resolving the diagnostic challenges in thyroid pathology requires a morphology-first approach. Identifying specific features, such as predominantly smooth luminal borders and the absence of nuclear pseudoinclusions, helps differentiate ITIs from metastatic PTC. Awareness that ITIs can exhibit atypical morphology is important, as misinterpretation carries significant clinical implications, particularly when intranodal thyroid tissue is incidentally discovered in the context of non-thyroid surgeries. Similarly, the “red flag” architectural patterns in a thyroid tumor, such as solid, cribriform, or micropapillary growth, should prompt the exclusion of metastatic disease. Immunohistochemical markers, including TTF-1, PAX8, and thyroglobulin, provide valuable diagnostic information but must be interpreted in the context of their known sensitivity and specificity for each tumor type. Careful morphologic assessment, judicious use of IHC, and correlation with clinical history enable accurate distinction between metastatic and non-metastatic lesions in thyroid pathology.

## Data Availability

No datasets were generated or analyzed during the current study.
